# Spontaneous Conception during In Vitro Fertilization prior to Embryo Transfer without the Opportunity for Preimplantation Genetic Testing

**DOI:** 10.1155/2019/1804948

**Published:** 2019-08-06

**Authors:** Brindha Bavan, Amin A. Milki

**Affiliations:** Stanford University, Department of Obstetrics and Gynecology, Division of Reproductive Endocrinology and Infertility, 1195 West Fremont Avenue, Sunnyvale, CA 94087, USA

## Abstract

In addition to the potential for multiple pregnancy, spontaneous conception during in vitro fertilization (IVF) can lead to undesired genetic outcomes. We present a case of a patient undergoing IVF with the intention of subsequent frozen embryo transfer after preimplantation genetic testing (PGT). Unprotected intercourse 6 days prior to egg retrieval resulted in a spontaneous pregnancy before the opportunity for embryo transfer. This case report highlights that spontaneous conception during IVF compromises the ability to transfer embryos that are euploid, unaffected by single gene disorders, or intended for gender balancing within a family when desired.

## 1. Introduction

Although IVF treatment for infertility is typically performed on patients that are thought to have a very small to a nonexistent chance of conceiving naturally, spontaneous conception following cessation of IVF has been reported in up to 29% of couples within 6 years following completion of treatment [1]. However, the probability of spontaneous conception occurring during any given IVF cycle is very small [2–4].

In a previous case report from 2001, we described the first known incidence of simultaneous spontaneous and IVF conception, which resulted in a quadruplet pregnancy [5]. As reviewed in this publication, intercourse during IVF treatment traditionally had been discouraged for the purpose of avoiding trauma to hyperstimulated ovaries and optimizing semen parameters. The cited patient's experience suggested that an additional benefit of avoiding intercourse during IVF cycles is mitigating the rare occurrence of a multifetal gestation and its related complications.

Here we describe a case report where spontaneous conception occurred during IVF treatment, prior to embryo transfer and without the opportunity for PGT. This patient's experience advocates that another benefit of abstaining from unprotected intercourse during IVF treatment is avoiding a spontaneous conception in cases where PGT is desired, particularly for those patients not willing to terminate for clinically serious genetic anomalies.

## 2. Case Presentation

A 32-year-old Chinese-Vietnamese nulliparous female presented to our clinic after trying to conceive for 1 year and 9 months without success. Her cycle length varied from 30 to 39 days. Body mass index was 19. Previous workup showed normal hormone levels and hysterosalpingogram. Antral follicle count was 16.

The patient's partner had normal semen analysis. Post-coital testing was reassuring with regard to mucous and presence of motile sperm.

The patient had previously undergone 3 cycles of letrozole and timed intercourse at an outside institution. She proceeded to undergo 4 cycles of letrozole and intrauterine insemination in our clinic, which were unsuccessful.

The decision was then made to move forward with IVF with embryo freezing after biopsy for aneuploidy screening. For her stimulation protocol, she began with 75 units of human menopausal gonadotropin and 125 units of recombinant follicle stimulating hormone, which was subsequently increased to 300 units. An antagonist was started on stimulation day 8. hCG trigger was administered on stimulation day 11, at which point the patient was found to have 15 follicles, 11 of which were larger than 12 mm. Oocyte retrieval occurred 2 days later where all follicles were aspirated, and 10 oocytes were obtained. Conventional IVF resulted in 4 embryos that were frozen.

The patient experienced vaginal bleeding 13 days after oocyte retrieval, which she believed to be a “heavier than usual period” lasting 4 days. She had a positive home pregnancy test about 29 days afterward, however. The patient had not yet undergone embryo transfer. Beta hCG level was 154,224 mIU/mL. Pelvic ultrasound 45 days after oocyte retrieval confirmed an intrauterine pregnancy with positive fetal cardiac activity consistent with 8 weeks 1 day gestation, suggesting conception around the time of oocyte retrieval. The patient and partner recalled having had intercourse 6 days prior to oocyte retrieval – at the cut off of when our program instructs patients to avoid intercourse, i.e., after the seventh day of stimulation. She proceeded to have an uncomplicated vaginal delivery of a healthy female newborn at 39 weeks' gestation weighing 3.09 kg. A case summary is provided in supplementary Figure 1.

## 3. Discussion

In recent years, preimplantation genetic testing has become increasingly popular and is performed in a significant portion of IVF cycles in many clinics. According to Society for Assisted Reproductive Technology National Summary preliminary data from 2016, 31% of IVF cycles involved PGT [6]. This trend has continued to grow. In our clinic, PGT was utilized in 43% of IVF cycles in 2016 and currently is used in the majority of cycles. In such cases, patients are provided with the opportunity of transferring euploid embryos and/or unaffected embryos in cases of single gene disorders. In these situations, conceiving with an untested embryo may have detrimental consequences.

This case report describes the unusual finding of a patient who spontaneously conceived during an IVF cycle. This clinical case introduces the benefit of recommending abstinence from unprotected intercourse during IVF treatment in order to prevent spontaneous conception without the opportunity to perform chromosomal analysis and/or single gene disorder screening of the transferred embryo if desired. It is possible that oocytes can be missed despite careful attempts to harvest all of them during the retrieval process and that unprotected intercourse can lead to spontaneous conception from sperm that could have survived in the female reproductive tract for several days.

This is particularly important in cases where patients would not choose to terminate a pregnancy due to a genetic anomaly with high morbidity and/or mortality leading to major impacts on quality of life for the patient, partner, and future child. Such an outcome is also a missed opportunity for those patients hoping to utilize PGT to enable gender selection for family balancing purposes.

Additionally, as in the previously discussed case report of simultaneous spontaneous and IVF conception resulting in quadruplet pregnancy, there is risk for multifetal gestation in patients having unprotected intercourse during IVF cycles, which leads to pregnancies at risk of complications such as preterm labor, preterm birth, preeclampsia, and placental abnormalities [7, 8]. This risk is further evidenced by reports of dizygotic twinning after single blastocyst embryo transfer (see supplementary Table 1).

For these reasons, it may be worthwhile to caution IVF/ICSI patients against unprotected intercourse after initial ovarian hyperstimulation if they have patent fallopian tubes, if partners have normal semen parameters, if PGT is desired, and if they will not consider reduction for an inherited genetic condition or high-risk multifetal gestation.

## Supplementary Materials

Supplementary Materials Figure 1: Summary of case presentation. Table 1: Literature review of dizygotic twinning after single embryo transfer.
