# Supplementary material for: Spontaneous Conception during In Vitro Fertilization prior to Embryo Transfer without the Opportunity for Preimplantation Genetic Testing
Source: Case Rep Obstet Gynecol. 2019 Aug 6;2019:1804948. doi: 10.1155/2019/1804948 (PMC6699353; doi:10.1155/2019/1804948)
Supplement: Supplementary Materials — Figure 1: Summary of case presentation. Table 1: Literature review of dizygotic twinning after single embryo transfer. [file 1804948.f1.pdf]

Supplementary Materials:

Figure 1: Summary of case presentation

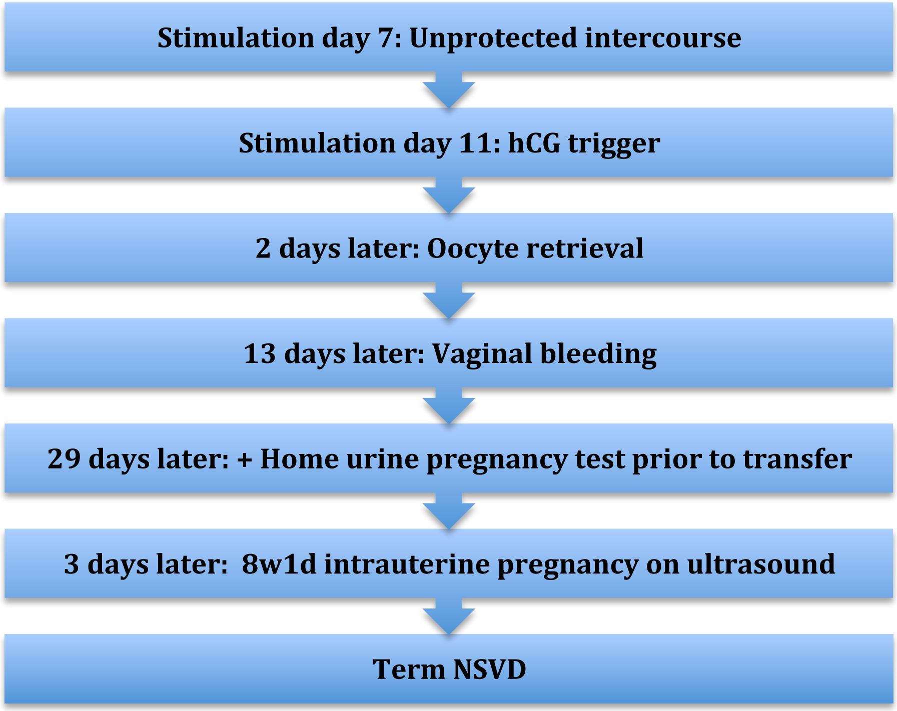

Table 1: Literature review of dizygotic twinning after single embryo transfer

Publication	Year	Lead Author	Study Type	Finding
Fertility & Sterility	2018	Vega[9]	Retrospectice cohort	23 out of 255 twins after sET[a] were dizygotic based on sex discordance
Human Reproduction	2014	Osianlis[10]	Retrospectice cohort	12 out of 109 twins after sET were dizygotic based on sex discordance
Journal of Assisted Reproduction & Genetics	2014	Takehara[11]	Case report	Dizygotic twins after sET based on sex discordance
Fertility & Sterility	2011	van der Hoorn[12]	Case report	Dizygotic twins after sET confirmed by different HLA typing
Human Reproduction	2010	Sugawara[13]	Case series (n=2)	Dizygotic twins after sET based on sex discordance
Journal of Assisted Reproduction & Genetics	2009	Kyono[14]	Case report	Dizygotic twins after sET based on sex discordance
Human Reproduction	2003	Cahill[15]	Case report	Quadriamniotic/quadrichorionic IUP[b] after transfer of 2 embryos confirmed by genetic testing
Reproductive Medicine & Biology	2001	Milki[5]	Case report	Quadriamniotic/quadrichorionic IUP after transfer of 2 embryos confirmed by genetic testing

[a] Single embyro transfer

[b] Intrauterine pregnancy
